# Approach to the diagnosis and management of dysbiosis

**DOI:** 10.3389/fnut.2024.1330903

**Published:** 2024-04-19

**Authors:** Kannayiram Alagiakrishnan, Joao Morgadinho, Tyler Halverson

**Affiliations:** ^1^Division of Geriatric Medicine, Department of Medicine, University of Alberta, Edmonton, AB, Canada; ^2^Kaye Edmonton Clinic, Alberta Health Services, Edmonton, AB, Canada; ^3^Department of Psychiatry, University of Alberta, Edmonton, AB, Canada

**Keywords:** microbiota, microbiome, dysbiosis, gut biotics, virtual organ

## Abstract

All microorganisms like bacteria, viruses and fungi that reside within a host environment are considered a microbiome. The number of bacteria almost equal that of human cells, however, the genome of these bacteria may be almost 100 times larger than the human genome. Every aspect of the physiology and health can be influenced by the microbiome living in various parts of our body. Any imbalance in the microbiome composition or function is seen as dysbiosis. Different types of dysbiosis are seen and the corresponding symptoms depend on the site of microbial imbalance. The contribution of the intestinal and extra-intestinal microbiota to influence systemic activities is through interplay between different axes. Whole body dysbiosis is a complex process involving gut microbiome and non-gut related microbiome. It is still at the stage of infancy and has not yet been fully understood. Dysbiosis can be influenced by genetic factors, lifestyle habits, diet including ultra-processed foods and food additives, as well as medications. Dysbiosis has been associated with many systemic diseases and cannot be diagnosed through standard blood tests or investigations. Microbiota derived metabolites can be analyzed and can be useful in the management of dysbiosis. Whole body dysbiosis can be addressed by altering lifestyle factors, proper diet and microbial modulation. The effect of these interventions in humans depends on the beneficial microbiome alteration mostly based on animal studies with evolving evidence from human studies. There is tremendous potential for the human microbiome in the diagnosis, treatment, and prognosis of diseases, as well as, for the monitoring of health and disease in humans. Whole body system-based approach to the diagnosis of dysbiosis is better than a pure taxonomic approach. Whole body dysbiosis could be a new therapeutic target in the management of various health conditions.

## 1 Introduction

Microbial cells involve different microorganisms like bacteria, fungi, protozoa, and viruses, which maintain balance in the microbial environment (1). The human body contains both human and microbial cells and as such, “Human beings are now considered as hybrid organisms” (2). Microbial communities inhabiting our body are known as the human microbiota. These microbiotas are seen in the skin, oral cavity, conjunctiva, respiratory tract, genitourinary (GU) and gastrointestinal (GI) tracts. The microbiota in different body surfaces has the ability to repel pathogens, a property known as colonization resistance (3). The microbiome is composed of the microbiota, its genes, and its products, which includes microbial structural products as well as microbial metabolites. It is the second genome of our body. Microbiome can be considered as an acquired invisible organ to the naked eye and present throughout the body. The human gut microbiome is made up of two or more microbiota that is organized to carry out a particular metabolic function and groups/ colonies of microbiome (within the body) with related function similar to an organ system. The connectivity of the microbiome is by integrating different microbiota such as eukaryote, prokaryote, archaea, and viruses and also includes host- microbiome interaction. Full metagenomic DNA sequencing is the basis of microbiome-based diseases (2). Understanding the entire view of the microbiome is not just learning about the colonies of microbiota, but also looking at the metabolic potential which can affect the microbial functioning including the host- microbiome interaction (4). This virtual organ has been a neglected organ till recently. Not only in Modern Medicine, but also in the traditional medicine like Chinese Medicine, Indian Medicine, like Ayurveda, are looking at the relationship of gut microbiota with host health and diseases. The holistic approach in traditional medicine is also now viewed in modern medicine with interplay of various organs with the spirit in the body. Most of these traditional systems give importance mainly to diet. By understanding the role of microbiome, we can appreciate the above-mentioned holistic concept in clinical practice with different medicinal systems (5, 6).

Non-pathogenic bacteria in the body have an effect on health. When there is microbial imbalance or compositional change, dysbiosis can result. Understanding the non-pathogenic microorganisms, microbial genes, and microbiota-derived metabolites will provide a more complete understanding of the microbiome. Different host factors affect the microbial environment (7). Microbiota composition varies with individual genotype, diet, and environment. Diet is the most important contributor of microbial flora. Microbiota plays a crucial role for energy extraction from nutrients through unique enzyme and biochemical pathways (8–10). The composition of the microbiome is host specific and changes throughout an individual’s lifetime (11). With environmental conditions especially with urbanizations, humans are exposed to different environmental exposures including pollution. Air pollutants like carbon monoxide (CO), nitrogen dioxide (NO_2_) which comes from vehicle exhaust and industrial wastes can play a role (12).

When dysbiosis occurs in the body, the pathogenic bacteria override the beneficial ones potentially causing diseases (13, 14). Whole body dysbiosis is a term to describe the changes in the quantity, variety, and/or location of microorganisms in the human body. This could include both intestinal tract dysbiosis and extraintestinal dysbiosis which have been linked to many human diseases. Malfunction of the microbiota virtual organ can affect even distant organs. The difficulty in explaining dysbiosis is due to the fact that there is no clear definition of a healthy gut microbiota with huge interindividual variation existing in the normal healthy population (15). In eubiosis, there is a preponderance of beneficial bacteria (Phyla Firmicutes and Bacteroidetes) over pathogenic bacteria (Phylum Proteobacteria) (16). Whereas in dysbiosis, changes in different components can be seen such as: (1) loss of beneficial bacteria, (2) overgrowth of potentially pathogenic bacteria, and (3) loss of overall bacterial diversity which can all occur simultaneously (15, 17).

## 2 Classification of whole body dysbiosis

The microbiome in the gut, skin, lung and genitourinary tract is quite distinct and plays a role in health and disease (16, 18) (Table 1). The whole body dysbiosis can be classified into 1. Gut microbial dysbiosis including oral dysbiosis and 2. Non-gut microbial dysbiosis.

**Table 1:** Conditions that exhibit an element of dysbiosis.

Gut vs. non-gut dysbiosis	Organ system	Associated diseases	Examples of altered populations of microorganisms**
Gut	Cardiovascular	Hypertension Dyslipidemia Atherosclerosis Atrial fibrillation Endocarditis	*Akkmermansia. muciniphila, Lactobacillus planterium, Lactobacillus rhmnosus* (19–21)
Non-gut	Respiratory	Asthma COPD Cystic fibrosis Pneumonia	*Streptococcus pneumoniae, Pseudomonas aeruginosa, Haemophilus influenza* (22) *Klebsiela, Moraxella, Haemophilus, Neisseria* (23)
Gut	Gastrointestinal	Irritable bowel disease Irritable bowel syndrome Gastroenteritis Non-alcoholic fatty liver disease Cirrhosis	*Roseburia* sp. *Feacalibacterium* sp. (24) *Bifidobacterium, Feacalibacterium* sp. (25) *Clostridioides difficile* (26) *Feacalibacterium* sp., *Coprococcis*, *Prevotella* (27, 28)
Non-gut	Genitourinary	Chronic kidney disease Bacterial vaginosis Pelvic inflammatory disease	*Bifidobacterium, Lactobacillaceae, Prevotellaceae* (29) *Neisseria gonorrhoeae, Chlamydia trachomatis* (30)
Non-gut	Central nervous systems	Meningitis Stroke/Cerebrovascular accident Parkinson’s disease	*Neisseria meningitides, Streptococcus pneumoniae* (31)
Gut	Psychiatric conditions	Dementia Depression Anxiety Bipolar disorder Schizophrenia	*Bacteroidetes, Actinobacteria, Prevotella, Bacteroides* (32, 33)
Gut/Non-gut	Oncological conditions	Gynecological cancers Colorectal cancer Skin cancers	Human papilloma virus (34) *Bacteroides fragilis, Escherichia coli, Enterococcus faecalis, Streptococcus gallolyticus* (35)
Gut	Autoimmune diseases	Rheumatoid arthritis Systemic sclerosis Sjogren’s syndrome Antiphospholipid syndrome	*Feacalibacterium* sp. (36)
Non-gut	Skin	Eczema Psoriasis Dermatitis	*Bifidobacterium, Bacteroides, Bacteroidetes* (37)
Gut	Endocrine or metabolic	Diabetes mellitus type 1 Diabetes mellitus type 2 Obesity	*Feacalibacterium* sp. *Escherichia* spp. (38) *A. muciniphills*, *Feacalibacterium* sp. *Bifidobacterium, Peptostreptococcus anaerobius* (39)

**Examples of microorganisms listed are not specifically associated with the listed diseases.

### 2.1 Gut microbial dysbiosis including oral dysbiosis

Huge colonies of microbiota reside within the gastrointestinal tract and produce metabolites which can enter into the blood circulation and affect extraintestinal organs (40–42) Dysbiosis of the oral microbiome is commonly seen with gingivitis, periodontitis, dental caries and oral candidiasis and is associated with systemic diseases (40).

In general, commensal microbiota is very important in maintaining health (43). The role that commensals and pathobionts play in their interaction with the microbial dysbiosis and host is so critical to shifts from health to disease in the oral cavity (44).

Imbalance of oral microbiome is related to disease states. The studies done with saliva showed decreased levels of pyruvate and N-acetylglucosamine in chronic periodontitis (45, 46).

Also, with aging, oral microbiome transformation occurs and lead to systemic diseases. After the age of 60 years, genus *Lactobacillus* can increase in the oral microbiome and is suspected to contribute to neurodegenerative disorders (47). A study by Jo et al. identified a distinctive connection between the oral and gut microbiota through lactobacilli, which might lead to functional alterations of the Parkinson Disease (PD)-associated microbiome (48).

#### 2.1.1 Changes occurring with dysbiosis in the gut

##### 2.1.1.1 When the normal gut microbiota becomes pathogenic with loss of beneficial bacteria

Normal gut microbiota may act like opportunistic pathogens, when host resistance fails by a gut infection or when the immune resistance becomes deficient. Gut bacteria are less abundant in the stomach and upper intestine and become more populated in the lower GI tract. Both gastric acid and bile have antibacterial properties and prevent pathological bacterial colonization in the upper GI tract. In addition, mechanical factors like peristalsis and the presence of antibacterial substances like bacteriocins and fatty acids also prevent pathological adherence. Antibiotics can inhibit or kill the normal microbiome, leading to pathological overgrowth resulting in dysbiosis (49). With a decrease in peristalsis and lower oxidation–reduction potential, higher numbers of gut bacteria are seen in the ileum and colon. The majority of colonic bacteria are obligate anaerobes (50), however, there are many facultative anaerobes, such as the Enterobacteria, that can contribute to significant negative metabolomic changes.

##### 2.1.1.2 Overgrowth of potentially pathogenic bacteria/loss of overall bacterial diversity representing the microbial signature of dysbiosis

There are more than 1,000 different species of microbiota in the gastrointestinal tract. Most of them are good and essential for optimum health such as *Bifidobacterium* and *Lactobacillus*. The gut microbiome is predominantly composed of Firmicutes, Bacteroidetes, Actinobacteria, and Proteobacteria. When there is an imbalance of either of these phyla of bacteria, dysbiosis can result. The typical signature of dysbiosis is the expansion of Proteobacteria (51). However, even certain Firmicutes, such as *Ruminococcus gnavus* and *Bacteroides fragilis* from the Bacteroidetes phyla have been found to play a role in bacterial dysbiosis, which can be seen if Inflammatory Bowel Disease (IBD) and Crohn’s disease (52). Bacteria that display pathogenic properties are referred to as pathobionts, and individual may be naturally colonized with these types of bacteria. Some examples of pathobionts include *Clostridioides difficile* (formally *Clostridium difficile*), *Enterococcus faecalis* and *Campylobacter* are considered as harmful and pathogenic. The above organisms have a relatively small infective doses, *C. difficile* at less than 10 spores, *E. faecalis* at 10 colony-forming units (CFU), and *Campylobacter* at 500–800 CFUs, that can lead to a disruption of the normal gut microbiome. With dysbiosis, two variations can occur with human microorganisms. 1. An abundance of good bacteria can raise the pH and lead to uncomfortable symptoms of gas, bloating and/or diarrhea. 2. With an abundance of bad bacteria, good bacteria can get diminished with loss of entire species that were present which leads to a reduction in the variety of organisms present (microbial diversity). This abundance of bad bacteria can cause widespread health concerns with depressed immune function, as well as an increase in inflammatory responses. In critical illness, “severe reduction in “health-promoting” commensal intestinal bacteria (such as Firmicutes or Bacteroidetes) and an increase in potentially pathogenic bacteria (e.g., Proteobacteria like *Salmonella, Vibrio*)” can occur (51). Gut bacteria dynamics vary based on location and the surface within the gastrointestinal tract. Penetration of pathogenic bacteria like *Shigella, Salmonella,* and *Campylobacter* throughout the gut surface needs a large bacterial exposure to cause illness (53).

### 2.2 Non-gut microbial dysbiosis

Outside of the gastrointestinal tracts, there have been other body systems that have been associated with microbial dysbiosis:

#### 2.2.1 Lung dysbiosis including ear, nose, throat tract dysbiosis

The lung microbiota commonly seen in healthy human lungs are Firmicutes, Proteobacteria, Bacteroidetes and Actinobacteria (54). With dysbiosis, altered microbial patterns are seen in the lungs (55). Evidence of microbial dysbiosis is seen with both ears, nose, throat (ENT) and lung conditions (56). Lung dysbiosis occurs with asthma, chronic obstructive pulmonary disease (COPD), idiopathic pulmonary fibrosis as well as lung cancers through the gut-lung axis (57–63).

Cigarette smoking is a known risk factor for COPD and lung cancer, and studies have shown that it can affect the microbiota both in the gut and lungs (64, 65).

A prospective cohort study by Szmidt et al. of 35,339 Swedish women found long-term (10 years) of high fiber intake (from cereal and fruit but not vegetable sources) to be linked with a 30% lower risk of COPD (66). A meta-analysis study showed daily consumption of 10 grams of dietary fiber, cereal fiber and fruit fiber reduced the risk of developing COPD but that effect is not seen with vegetable fiber (67).

The growing evidence points out that gut microbiota can influence the lung microbiome systemically through the gut-lung axis. This opens the potential for intranasal aerosol microbiota therapy in lung dysbiosis subjects (68).

The lung microbiome has been showed to play a role in the progression of lung cancer or its risk for recurrence (69).

#### 2.2.2 Skin dysbiosis including conjunctival and eye dysbiosis

Skin is the largest organ and the skin microbiome contributes to immunity. Microbial composition varies with dry, moist, or oily areas of the skin (70). Recent evidence supports the association between ocular diseases and gut microbiota through gut-eye axis (71). Skin dysbiosis occurs through the skin-gut axis (72), which can be affected by diet (71). Western diet (high fat, higher amount of sugar and salt and processed food ingredients) has been related with psoriasis and atopic dermatitis. Dermatitis herpetiformis associated with celiac disease has shown improvement when changed to a gluten- free diet (73). Ultraviolet B exposure which increases the serum Vitamin D levels by altering the alpha and beta diversity of the gut microbiome (73).

In addition to probiotics (74), prebiotics can also help with skin conditions. An extract from Probiotic *Lactobacillus planatarum* helps in the management of acne lesions by improving skin barrier function (75). A combination of a probiotic and prebiotic like *Bifidobacterium* and Glucooligosaccharides (GOS) reduce the transepidermal water loss of skin and prevent erythema (76). GOS have been used in many skin conditions like atopic dermatitis, eczema and photo aging diseases (77). Metabolites produced by probiotics (also known as postbiotics), like sodium butyrate, are used to treat psoriasis which is a proliferative skin disease (78). Other postbiotics, like short chain fatty acids (SCFA), produce anti-inflammatory activities in various skin disorders (76). Through the gut-skin axis, gut dysbiosis is associated with skin conditions, such as atopic dermatitis, psoriasis, acne and rosacea (79–81). Microbials metabolites can affect the immune system via the gut-skin-axis (73). Imbalance of the microbiome can increase the chance of skin infections and diseases, whereas restoring balance with a healthy microbiome may help in the recovery of skin diseases including wound healing (82, 83). Skin cancers can happen because of the microbial toxins causing cellular damage (84).

Microbiota with distinct characteristics is seen in the gut and skin, and this specific microbial composition is affected by a range of other individual attributes, such as age, ethnicity, genetics, climate and skincare (85, 86). Aging, diabetes and skin diseases, can cause microbial dysbiosis and increase infection risk (87).

#### 2.2.3 Genitourinary dysbiosis

*Lactobacillus* is a common microbiota seen in the healthy vagina (88). With menopause there is reduced estrogen which can increase the vaginal pH, altering the vaginal microbiome and lead to reduced levels of this genus of bacteria. La*ctobacillus* prevents proliferation of pathogenic microorganisms/vaginal dysbiosis. Postmenopausal changes in the gut microbiome are associated with increased short-chain fatty acids and hydrogen sulfide levels and may play a role in the gut -vagina- bladder- axis. *Lactobacillus* function to protect the vaginal mucosa against the colonization and proliferation of pathogenic microorganisms. Urinary microbiome dysbiosis is associated with interstitial cystitis, urinary tract infection (UTI), bladder pain syndrome and different types of urinary incontinence (29, 88–94).

Microbiome in the urinary and the genital tract may arise from the gut or vaginal microbiota in females as well as from the environment (95).

## 3 Concept of whole body dysbiosis

**Figure 1:**
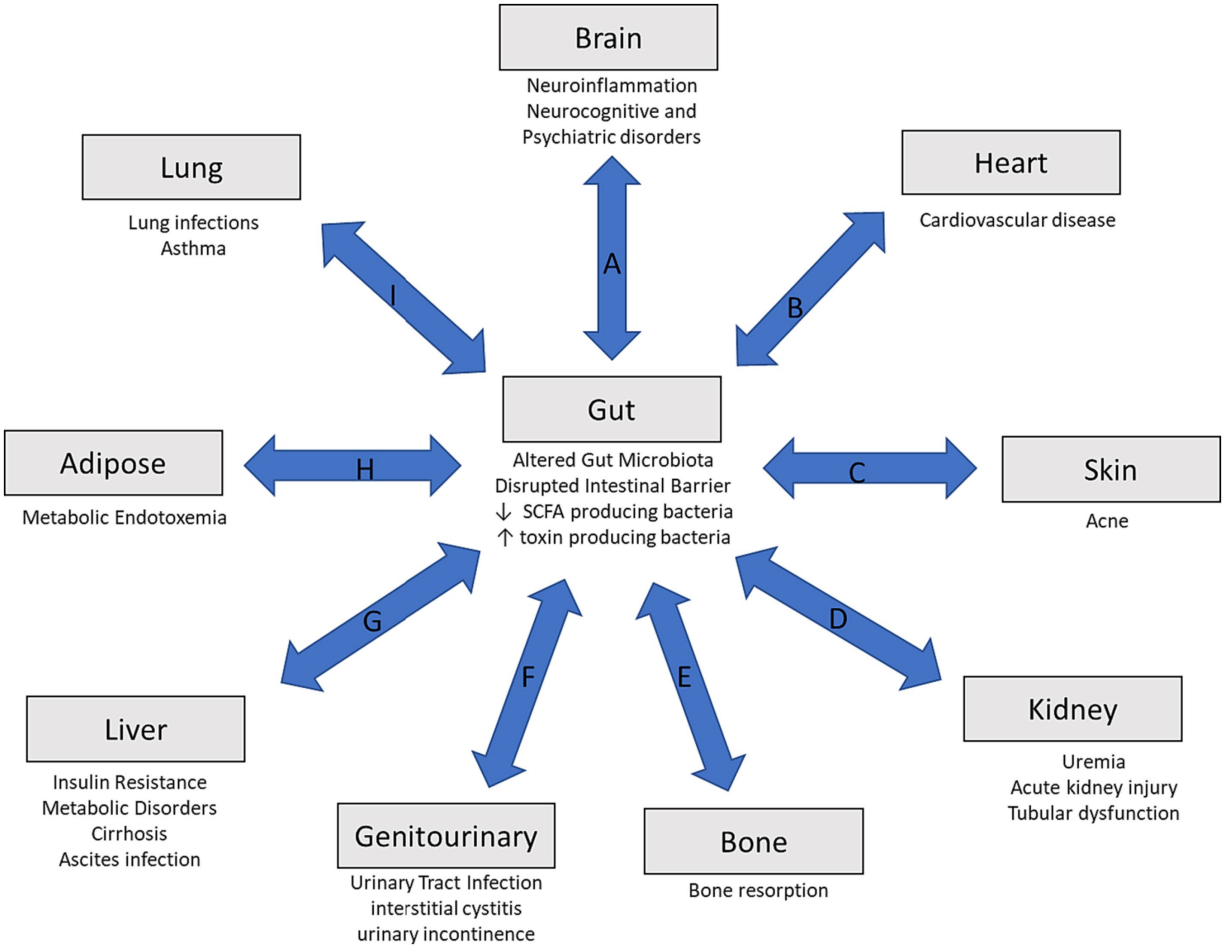
Overview of the gut-organ axis. Disruptions in the gut microbiome can lead to a decrease number of short-chain fatty acid producing bacteria and increased toxin producing bacteria. Along with this, there may be disruptions in the intestinal barriers leading to bacterial translocation that may influence systemic inflammation. (A) The gut-brain axis, (B) gut-heart axis, (C) gut-skin axis, (D) gut-kidney axis, (E) gut-bone, (F) gut-genitourinary axis, (G) gut-liver axis, (H) gut-adipose, and (I) gut-lung axis.

Various extraintestinal organs play a role in the physiological function of the gut microbiome. Gut and non-gut dysbiosis communicate through different axes in a bidirectional manner. This highlights the concept of the gut–organ axis (Figure 1) (11, 96). When there is a disruption in the gut microbiome, there can be a reduction in SCFA producing bacteria along with an increase in toxin producing ones. The intestinal barrier can also be weakened, contributing to bacterial translocation that can influence systemic inflammation. This has been associated with various health conditions, such as diabetes, cardiovascular disease, and neurocognitive disorders (Figure 1). Immune signaling and metabolic reactions contribute to these pathways. This interrelationship can lead to various diseases. Interplay between gut and non-gut dysbiosis, and as such, the different axes is shown in Figure 1. This has opened a new concept, and we coin a new terminology called “whole body dysbiosis.” This concept should help to better understand the pathogenic links between different organs and different medical conditions. (A) The Gut-Brain axis may be affected by dysbiosis leading to altered levels of neurotransmitters and bacterial metabolites. Bacterial translocation can influence neuroinflammation which may play a role in neurocognitive and psychiatric conditions. (B) Gut-Heart Axis may be impacted by altered bacterial population that may generate increased levels of trimethylamine (TMA) that is oxidized in the gut to trimethylamine-N-oxide (TMAO) that can increase the risk of cardiovascular disease. (C) Gut-Skin Axis can be influenced by increases immune response from bacterial translocation which results in increased sebum leading to skin disorders, such as acne. (D) Gut-Kidney Axis can be influenced by dysbiosis by increased toxin forming bacteria leading to uremic toxins damaging the kidneys. The production of TMAO can also contribute to renal insufficiency. (E) Gut-Bone Axis can be impacted by increased immune response and signaling that can affect bone resorption. (F) Gut-Genitourinary Axis is influenced by changes in the gut microbiome can lead to increased SCFA and hydrogen sulfide levels. Postmenopausal reduction in estrogen leads to increased vaginal pH causes a decrease in *Lactobacillus*. This in turn can contribute to urinary tract infections (UTI), interstitial cystitis, and different type of urinary incontinence. (G) Gut-Liver Axis can be influenced by bacteria metabolites through the hepatoenteric circulation which can activate hepatic stellate and Kupffer cells leading to cytokine and chemokine production resulting in liver damage, insulin resistance, and metabolic disorders. (H) Gut-Adipose Axis can be affected through increased lipopolysaccharide exposure and causing metabolic endotoxemia. (I) Gut-Lung Axis is altered due to increased inflammation and immune signaling leading to conditions such as asthma.

## 4 Metabolic consequences of dysbiosis

Short chain fatty acids are the metabolic end products of bacterial fermentation, which may have an effect on host health. Short chain fatty acids like propionate, acetate, and butyrate affect carbohydrate fermentation and play a role in the regulation of intestinal motility, as well as, anti-inflammatory function with prevention of leaky gut barrier. Indole degradation of the amino acid tryptophan increases epithelial-cell tight-junction resistance and reduces inflammatory markers. The gut microbiome plays a role in different vitamin synthesis like Vitamin K2, B12, biotin, folate which are co-factors for various metabolic pathways. Ceramide induces degradation of sphingomyelin via alkaline sphingomyelinase and in the prevention of tumorigenesis. Ceramide also plays a role in the regulation of gut-liver axis (97).

## 5 Dysbiosis and different diseases

Dysbiosis has been associated with a growing list of diseases (Table 1) with complex pathologies. Dysbiosis occurs commonly in GI and non-GI diseases. Human microbiota is linked to different diseases including noncommunicable diseases and autoimmune diseases (33, 98–100). The microbiota may also play a role in cancer through immune modulation and activation of signaling pathways for cell proliferation (101–103). Under conditions of dysbiosis, there can be a reduction of protective bacteria with a switch to more abundant pathogenic and cancer-promoting bacteria, which can include *Streptococcus bovis*, *Sulfidogenic* bacteria, *Fusobacterium nucleatum*, *Bacteroides fragilis*, *Clostridium septicum*, *Escherichia coli, Helicobacter pylori*, *Enterococcus faecalis*, Human papilloma virus, John Cunnigham virus, and Epstein Barr virus. This can include the promotion of particular functions such as angiogenesis, loss or apoptosis, and cell proliferation (104). There have been other studies that have shown certain microorganism can potentially contribute to colorectal cancer via the production of toxic metabolites, interactions with the immune system, and the release of genotoxic virulence factors (105).

## 6 Risk factors for dysbiosis

**Figure 2:**
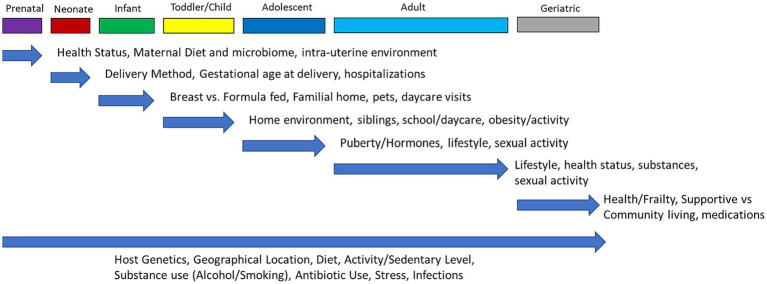
Dynamic changes of microbiome over the life span of humans. Throughout each stage of life (top row) there are factors that can have an influence and impact the human microbiome. Each blue arrow highlights what specific factors are most likely to alter the microbiome at that stage of life, with the effects potentially remaining throughout the rest of life. The bottom arrow indicates influences on the microbiome that are non-modifiable (host genetics) or that can occur throughout all stages of life.

Whole body dysbiosis could be a risk factor for many diseases. As shown in Figure 2, the first human microbiome is inherited at birth and is highly stable, whereas the acquired microbiome after birth depends on environmental factors (98). In some cases, studies have linked dysbiosis to being born via C-section and being formula fed among newborns (106).

### 6.1 Modifiable risk factors

Before birth, the fetus is considered to be sterile. Starting from birth, different modifiable risk factors like type of birth, breast feeding, antibiotic use, life style factors (dental hygiene, alcohol, smoking), environmental factors (air pollution) and also unprotected sex can influence the microbiome composition and diversity.

There are many factors that can lead to the state of dysbiosis, including the excessive or wrong use of antibiotics, excessive alcohol consumption, increased intake of sugar or protein, frequent use of antacids, exposure to pesticides on unwashed fruits and vegetables, and chronic stress with weakening of the immune system (48) Also, poor dental hygiene, unprotected sex, and anxiety can lead to dysbiosis. With sexual intercourse, there is emerging evidence that there is a transmission and exchange of the microbiome found in vaginal and seminal fluids (107).

The composition of our microbiota is influenced by host genotype, environment, lifestyle and diet (108). There is a dysbiosis risk stratification scale called INDIS survey which helps stratification of intestinal dysbiosis in adult patients (109).

## 7 Medications and dysbiosis

Most commonly, it is expected that when treating patients with antibiotics, there will be an impact on the gut microbiome. However, there have been studies that found other types of medications that can have antimicrobial effects (110). Since it is an exhaustive list, we have focused on selected medications to explain the dysbiosis effects of drugs. We have highlighted only some of the typically prescribed medications in older adults, such as antibiotics, proton pump inhibitors, metformin, psychotropics, statins, and opioids, were discussed with its affect on gut microbiome.

### 7.1 Antibiotics

In general, antibiotic treatment reduces the diversity of gut microbiota species, which leads to metabolic shifts, increases gut colonization, which can lead to bacterial antibiotic resistance (111–113) In humans antibiotic use is associated with Antibiotic-Associated Diarrhea, *C. difficile*-associated Diarrhea, *Helicobacter Pylori* Infection in the short term. Where as in the long term, antibiotic use can contribute to the development of obesity, asthma, allergy, and IBD (114, 115).

There have been various human studies that examined the impact of antibiotics on the gut microbiome (Table 2). Even a single exposure of antibiotic use, even in childhood, can have a lasting effect on gut microbiome, more so with broad-spectrum antibiotics. It is hypothesized that the use of antibiotic regimens, both single and multiple use, may influence mental health conditions, such as depression and Alzheimer’s dementia, by changing the population of the gut microbiome (32, 112).

**Table 2:** Exposure to antibiotics and health outcomes from selected human studies.

Antibiotic exposure	Type of study	Outcome	Study
Any exposure of lifetime	Population study	An increase risk of Schizophrenia (HR of 1.37 with 95% CI = 1.20–1.57) and affective disorders (HR 1.64 with 95% CI = 1.48–1.82)	(116)
Early life/childhood exposure	Population study	It was found that with any antibiotic exposure there was increased risk of mood disorders and ADHD (HR 1.15 with 95% CI = 1.13–1.17)	(117)
Exposure greater than 1 year from index diagnosis of either 1 of 7 classes of antibiotics	Nested case control study	Study examined patients with depression (*N* = 202,974), anxiety (*N* = 14,570), and psychosis (*N* = 2,690). Those treated with a single antibiotic course has a higher risk for depression with all antibiotic groups with AOR of 1.23 for penicillins (95% CI = 1.18–1.29) and 1.25 (95% CI = 1.15–1.35) for quinolones. There was increased risk with recurrent exposures of 2–5 courses (AOR 1.40 with 95% CI = 1.35–1.46) and > 5 courses (AOR 1.56 CI = 1.46–1.65) of penicillin. With anxiety there was an increased AOR with penicillins and sulfonamides of 1.17 (95% CI – 1.01-1.36) for a single course of penicillin and with >5 courses an AOR of 1.44 (95% CI 1.18–1.75) There was no observed change in risk for psychosis with any antibiotic group.	(118)
Antibiotic exposure either alone or in combination with other antibiotics or medications	Systematic review	There was an increased risk of developing depression by 20% in patients, even 5–10 years after use. Suspected that alteration in the microbiome and diversity was a contributing factor.	(119)
Early life/childhood exposure (within first 2 years of life)	Meta-analysis	Studies included up to 3.5 million children. Found that antibiotic exposure within the first 2 years lead to an increased risk of asthma, eczema, and obesity (*p* < 0.05). It was found that exposure during the first 6 months being most critical, as this is the time when the microbiome is more susceptible.	(120)
Cumulative antibiotic use of the course of life	Cohort study- NHS	The study looked at 36429 women and antibiotic use in young (20–39), middle (40–59), and late (≥60) adulthood. There was an increased risk of CVD in the late adulthood group with long-term (> 2 months) antibiotic use (HR 1.32, 95% CI 1.03–1.70). As well, longer duration of antibiotic use in middle adulthood group has higher risk of CVD (P trend = 0.003). No risk was observed in the young adult group.	(121)

AOR, adjusted odds ratio; CVD, cardiovascular disease; CI, confidence interval; HR, hazard ratio; NHS, nurses health study.

Different classes of antibiotics have been examined for their potential impact on the gut microbiome. Beta-lactam and glycopeptide, such as amoxicillin and ceftriaxone, have been found to cause dysbiosis in the gut (113–115) As well, changes in the community composition in the gut has been found to be caused by DNA replication inhibitors or DNA-damaging antibiotic, including quinoline and nitrofurantoin as examples (113–115). Alteration in mucus secretion, ion transport, and inflammatory response has been found related to the non-antimicrobial effects of macrolides. Other transcription and protein synthesis inhibitors have also been found to cause the distress of the gut microbiome network (113–115). With respect to the vaginal and urinary microbiome, there has been found a decrease in the overall diversity with increased abundance of *Lactobacillus iners* when exposed to nitroimidazole antibiotics, such as metronidazole and azithromycin (113–115). With different classes of antibiotics, changes in the gut microbiome can be seen, however, specific microbial changes are not consistently seen across studies (Table 3).

**Table 3:** Antibiotic treatment and its influence on the gut microbiome (122, 123).

Class of antibiotic	Antibiotic treatment	Impact on the gut microbiome	
		Increased	Decreased
Beta-lactam	Amoxicillin with clavulanic acid	*Enterobacteriaceae* *Parabacteroides distasonis* *Escherichia* *Enterobacter*	*Bifidobacterium* *Clostridium cluster XIVa* *Bacteroides fragilis* *Roseburia*
Beta-lactam	Imipenem	*Akkermansia muciniphilia*	Enterobacteriaceae Clostridia *Bacteroides* *Enterococcus*
Beta-lactam	Meropenem	*Enterococcus* *Bacteroides* Enterobacteriaceae Clostridia	
Beta-lactam and beta-lactamase inhibitor	Piperacillin and Tazobactam		Enterobacteriaceae *Bifidobacterium* *Eubacterium* *Lactobacillus*
Multiple	Amoxicillin with clarithromycin	Firmicutes	Bacteroidetes
Macrolide	Clarithromycin	*Bacteroides* *Proteobacteria*	*Actinobacteria* Firmicutes
Macrolide	Azithromycin		*Bifidobacterium*
Macrolide	Surotomycin	Bacteroidetes *Prevotella*	*Clostridium* *Lactobacillus* *Bifidobacterium* *Enterococcus* *Streptococcus* *Enterobacterium* *Bacteroides fragilis*
Multiple	Clarithromycin, metronidazole, and omeprazole	Firmicutes *Proteobacteria* *Enterococci*	*Actinobacteria* *Bifidobacterium* *Clostridium* *Bacteroides*
Glycopeptides	Vancomycin	Firmicutes*	*Lactobacillus* *Clostridium* Firmicutes*
Quinolone	Levofloxacin		*Escherichia coli*
Quinolone	Ciprofloxacin	*Bacteroides* *Enterococcus*	*Bifidobacterium* Enterobacteriaceae *Alistipes* *Faecalibacterium* *Oscillospira* *Ruminococcus* *Dialister*
Lincosamide	Clindamycin	*Clostridioides difficile* *Bacteroides* Lactobacillus* Clostridia	*Bifidobacterium* Lactobacillus* Enterococcus *Klebsiella* *Enterobacter* *Cirtrobacter*
Cephalosporin (2nd generation)	Cefprozil	*Lachnoclostridium bolteae*	*Bacteroides enterotype*
Cephalosporin (3rd generation)	Ceftazidime		Enterobacteriaceae *Lactobacillus*
Cephalosporin (4th generation)	Cefepime		*Escherichia coli* *Bifidobacterium*

*Have shown both an increase and decrease based on different study results. Adapted from Patangia et al. (122) and Yang et al. (123).

When the gut microbiome is exposed to antibiotics, the changes can persist from weeks to years. Broad spectrum antibiotics more commonly cause dysbiosis. It was found that treatment with ciprofloxacin, clindamycin, and clarithromycin with metronidazole left changes to the gut microbiome lasting 1, 2, and 4 years, respectively (113–115). It may be that the long-term consequences from antibiotic exposure may play a role in the development of obesity, allergies, and even asthma (113–115). At present, due to the heterogenicity of the study designs, there remains limitations on determining the effects of antibiotics on dysbiosis. Future antibiotic studies should control for medical comorbidities, age, and diet to get a better understanding of the impact of just antibiotics alone.

### 7.2 Proton pump inhibitors

One of the common medications used to treat gastroesophageal reflux and peptic ulcer disease are proton pump inhibitors (PPIs). However, there is evidence that these medications can contribute to dysbiosis, primarily through *Clostridioides difficile* infections, with higher rates found in hospitalized patients (124, 125). Studies showed that the use of PPIs can lead to a decrease in the alpha-diversity in those prescribed the medication compared to those not using them (125, 126). There have been multiple studies that found individuals using PPIs who had a significant increase in various bacterial genera, including, *Enterococcus*, *Streptococcus*, *Staphylococcus*, and *Rothia*; as well as, the species such as *Lactobacillus salivarius* and a potentially pathogenic species of *Escherichia coli* (127). Another study by Bruno et al. also found that PPIs can lead to dysbiosis throughout various segments of the GI tract with increased Enterobacteriaceae, Enterococcaceae, and Lactobacillaceae while there was a decrease in Ruminococcaceae and Bifidobacteriaceae in the colon (128). It is suspected that individuals who are on long-term PPI use can be at risk for enteric infection through dysbiosis which can lead to irritable bowel syndrome (IBS) development (129).

### 7.3 Metformin

It is a common medication, used in the treatment of diabetes. It has been found that patients treated with this medication, when compared to non-users, did not have a significant difference seen in the alpha-diversity, but rather some difference in beta-diversity (130). Another study also found that metformin can lead to an alteration in the gut microbiome, causing an increase in various bacteria, such as *Akkermansia muciniphila*, *Escherichia* spp*.,* and *Lactobacillus*, while other bacteria, like *Intestinibacter* were found to have decreased levels (131). This study also highlighted that metformin can promote SCFA production, which has been found to help support the intestinal barrier and regulate the secretion of gut peptides (131). A study by Wu et al. found that metformin can exhibit a positive influence on the gut microbiome and when fecal samples of metformin-treated individuals were transferred to germ-free mice, the mice had improved glucose tolerance (132).

#### 7.3.1 Antidepressants

Chronic exposure of antidepressants in preclinical studies have shown a decrease in richness of gut bacteria compared to controls (133). This study, by Lukić et al., included mice treated for 21 days with either fluoxetine (10 mg/kg), escitalopram (10 mg/kg), venlafaxine (10 mg/kg), duloxetine (10 mg/kg), or desipramine (20 mg/kg) and found that all these antidepressants, except desipramine, lead to a reduced richness of the gut microbiome (133). The authors also looked at the genus *Ruminococcus* and duloxetine and found that mice treated with the antidepressant along with a supplementation of the *R. flavefacians*, showed an attenuation of the antidepressant effects (133). When looking at the gene expression, *R. flavefaciens*, was found to decrease the expression of synaptic signaling and neurodegenerative genes, similar to that of patients with depression (133). A study by Cussotto et al. found that mice given fluoxetine had an inhibited growth of *Succinivibrio* and *Prevotella* (134). In humans, it has been found that treatment with antidepressants can affect the composition of the gut microbiome (135). Among the selective serotonin reuptake inhibitors, the antidepressant sertraline has been found to have the most potent antimicrobial activity and even a synergistic effect with antibiotics (136).

#### 7.3.2 Antipsychotics

There are research studies looking at the influence of antipsychotics on the gut microbiome in both animals and humans. It was found that germ-free mice treated with olanzapine did not exhibit the same weight gain as their colonized counterparts. When these mice were colonized, the weight gain was then seen, suggesting that the gut microbiome may be involved and play a role with the side effect of olanzapine (137). In a human study involving patients with schizophrenia, when treated with risperidone for 24 weeks, there was a change in the gut composition that included increased Bifidobacterium and *Escherichia coli,* with decreased *Clostridium coccoides* and *Lactobacillus* (138). It was also found that female patients treated with atypical antipsychotics had decreased species diversity with Lachnospiraceae, *Akkermansia*, and *Sutterella*, compared to those treated with non-atypical antipsychotics; interestingly male patients did not show a significant diversity difference (139). It appears that patients treated with antipsychotic showed an altered ratio of Firmicutes: Bacteroidetes, resembling that seen in obese patients, which may provide evidence to the associated weight gain seen with these medications (140).

### 7.4 Non-steroidal anti-inflammatory

There have been various studies looking at the impact of NSAIDs medications on gut bacteria through dysbiotic changes. Specific NSAIDs, such as celecoxib and ibuprofen lead to an increase in certain bacterial families such as Enterococcaceae, Enterobacteriaceae, Erysipelotrichaceae, Acidaminococcaceae, and Desulfovibrionaceae (141). In elderly patients who are prescribed NSAIDs a depletion in *Lactobacillus* and *Collinsella aerofaciens* and an enrichment in *Roseburia* is seen, compared to non-users (142).

### 7.5 Opioids

Opioids have been commonly prescribed to treat moderate to severe pain. Studies have found that they may play a role in bacterial translocation through disruption of the gut barrier (143). When examining hospital patients, opioid use was associated with increased alpha-diversity, particularly with *Parabacteroides*, *Propionimicrobium*, *Alistipes*, *Sutterella*, *Clostridium*, *Bifidobacterium*, unclassified Lachnospiraceae, and *Pyramidobacter*; with a negative association with Polyomavirus, *Pseudomonas*, unclassified Ruminococcaceae, *Candida*, and *Megamonas* (144). It is hypothesized that since opioids tend to delay GI transit time, this may be more conducive to bacterial growth in the colon and allow for the increased diversity seen in certain microbial populations.

### 7.6 Statins

Statins are medications that are commonly and routinely used to help treat dyslipidemia that often include some GI side effects. There has been evidence that the use of statins can contribute to changes in the beta-diversity of the gut microbiome (145). In a study looking at idiopathic Parkinson patients, the use of statins leads to an increased relative abundance of Burkholderiaceae, Propionibacteriaceae, Enterococcaceae, Actinomycetaceae, and Enterobacteriaceae (146). As well, viruses were found to be increased in participants that were treated with a statin (146). Variation in statin response has been attributed to the effect of microbiota (147). However, when controlling for statin exposure, no significant difference was observed between the participants and controls, which was felt to be due to a small sample size. A study looking at human subjects found that individuals treated with rosuvastatin for 4–8 weeks had a significantly altered gut microbiome (148). In particular, the phyla Firmicutes and Fusobacteria showed a negative correlation to the lowering of the low-density lipoprotein cholesterol (LDL-C) level while Cyanobacteria and Lentisphaerae were positively associated with the lower LDL-C level (148).

In conclusion, the end results of these medication induced microbiome alterations provide a significant impact on dysbiosis and contributes to many diseases.

## 8 Clinical features of dysbiosis

Most patients with dysbiosis present with gastrointestinal symptoms like halitosis or bad breath, frequent flatus, bloating, food intolerances, food sensitivity, abdominal cramping, diarrhea and/ or mucus in the stool. They can have other symptoms like vaginal or rectal itching, skin conditions, fatigue, mood symptoms like depression or anxiety, and problems with memory. These symptoms depend on the system impacted by dysbiosis (149–151).

## 9 Investigations for dysbiosis

Dysbiosis cannot be diagnosed through standard blood tests or through scopes (endoscopy or colonoscopy), but many tests (Table 4) may aid with diagnosing dysbiosis, which is not commonly done in clinical practice at this point. Generally, it is known as CDSA (Comprehensive Digestive Stool Analysis).

**Table 4:** Diagnostic tests for dysbiosis.

Test	Description
Stool test	This test can help determine the overall balance of bacteria and present of yeast. Through the use of Polymerase Chain Reaction (PCR) it can determine the ratio of Firmicutes to Bacteroidetes along with the presence of *Lactobacillus* and *Bifidobacterium* (152).
Diversity of the microbiota (dysbiosis indexes)	These indexes help to determine between intestinal microbial communities. Often alpha and beta diversity assessments are commonly used and should be interpreted based on the context of clinical findings (153). Alpha-diversity was calculated using the Shannon index depending on the gene and species profile.
Urine test	Look for microbial metabolites in the urine using Nuclear magnetic resonance (NMR) (154)
Intestinal permeability assessment or mannitol-lactulose intestinal permeability test	This test can explore intestinal permeability and dysbiosis and suggest leaky gut syndrome. An individual can will consume the sugars mannitol and lactulose, if there is permeability in the gut, these guts will be detected in the urine at elevated levels (155).
Hydrogen or methane breath test	A baseline breath gas measurement if first done and the followed by the patient ingesting a standardized substrate solution (typically lactulose) that is indigestible by humans but easily digestible by bacteria. Next, the breath of the individual is measured every 20 min to assess the amount of hydrogen and methane. These readings will determine the degree of microbial fermentation within the upper GI tract. A positive indication of dysbiosis is confirmed with rapid and steady rises of the hydrogen and methane readings. Repetition of this test can be used to gauge treatment progress of a leaky gut (156, 157).
Large scale bacterial marker profiling	This method of identification used various specific markers on species/taxa of bacteria. One example is the use of 54 probes that target the 16S rRNA gene at different bacterial taxonomic levels (covering Firmicutes, Proteobacteria, Bacteroidetes, Actinobacteria, Tenericutes, and Verrucomicrobia). This is known as the GA-Map dysbiosis test. When classifying a sample, it is compared to a reference population, a score of 1 to 5 is used, where a recording of greater than 2 is considered dysbiosis. It can also look at targeted species and give a score of −3 to 3 where negative values suggest a reduced abundance and positive values suggest increased abundance (153).
Relevant taxon-based methods	Other types of dysbiosis indexes have been developed to look at specific taxa and with the goal of being more simplistic and easily interpreted. These indexes are calculated based on ratios between abundance (153).
Neighborhood classification	This technique is used to measure the microbial dysbiosis in an individual compared to a healthy control. This is determined by quantifying the deviation a specific sample is from a reference sample set using dissimilarity matrices (153).
Random forest prediction	Through the use of a machine learning, algorithm random forest and a generated dysbiosis index based on operational taxonomic units examining abundances normalized by GMPR (geometric mean of pairwise ratios). It uses a range from 0 to 1, where values approaching 1 suggest a high likelihood that the gut microbiota is from a symptomatic individual (often used in small intestine overgrowth (SIBO) patients) (153).
Combined alpha and beta diversity	This method is most commonly used in sequencing-based microbiota studies that provide a general description of microbial communities. Alpha is use to describe the number of unique taxa (richness) and their distribution (evenness) within a community and is often considered a biomarker of health. Beta is used to assess difference in community composition between individuals, or can be applied when assessing patients versus healthy controls. There is a combined method described as a dysbiosis index that uses a range of 0–5, where values greater than 1 suggest dysbiosis (153).
Oral carnitine challenge test	This test was designed to help determine and apply personalized nutrition to an individual based on the function of their gut microbiome. This method considers the gut microbiome as a “bioreactor” and it is provided inputs in the form of fermentable materials and the outputs (microbial byproducts) are measured either in the blood or urine. This test can also be used to measure metabolites from microbial fermentation (158)
Gut dysbiosis biomarkers	There are certain biomarkers that may give an indication of gut dysbiosis. Certain gut microorganisms are able to release urolithins (anti-inflammatory metabolites) when exposed to dietary polyphenols. These metabolites may serve as biomarkers of gut microbiota composition and functionality (159). Other biomarkers that have been studied for metabolite profiling and diagnosing dysbiosis include, trimethylamine-N-oxide, short-chain fatty acids, 3-indoxyl sulfate, p-cresyl sulfate, secondary bile acids, hippurate, human β-defensin-2, chromogranin A, secreted immunoglobulins and zonulin (160).
Dysbiosis indexes	Dysbiosis can be determined and quantify by relevant taxon-based methods, bacterial marker profiling, alpha and beta diversity. At this time, these indexes may be used as a diagnostic marker of dysbiosis, but are not predictors of a disease or disease process (153).

### 9.1 Comprehensive digestive stool analysis

CDSA include analysis of different microbiota such a lactobacilli, bifidobacteria, *E. coli*, Proteus, *Pseudomonas*, *Salmonella*, *Shigella*, Vibrio, yeast and microbiome analysis including sequencing technologies, dysbiosis indexes, metagenomics, metatranscriptomics as well as assessment of microbial metabolites like Short Chain Fatty Acids. It also includes Hydrogen Breadth test, which detects the presence of gases produced by bacteria and excessive gases indicate imbalance of bacteria (161, 162).

Stool or fecal specimens can be used to look at gut microbiota and microbiome because of relative ease of access of the sample (152).

Sequencing technologies are usually based on samples collected from inner—colonic (mucosal biopsy/capture microdissection, luminal brushing, intestinal fluid lavage), which gives a better view of the colon’s microbial diversity. 16S ribosomal RNA (rRNA) amplification and whole-genome shotgun sequencing (WGS), are the two typical sequencing technologies used to diagnose gut microbiota diversity (152).

#### 9.1.1 Dysbiosis indexes

Microbiome analysis using metrics of markers of dysbiosis included alpha-diversity and beta-diversity as well as distributions of predominant phyla. The three alpha-diversity indices (Shannon index, Simpson’s Index, Chao-1 Index) and beta- diversity metrics like Bray-Curtis distance will be done. Alpha diversity, which indicates the relative abundance of microbial species in a biological sample, where as beta diversity and gamma diversity measures species diversity over time (165) Dysbiosis indexes have to be interpreted in the context of the clinical findings (166). Dysbiosis is measured by using dysbiosis indexes. To quantify dysbiosis, large-scale bacterial marker profiling, relevant taxon-based methods, neighborhood classification, random forest prediction, and combined alpha and beta diversity indexes are used (166). Studies using these indexes showed among chronic respiratory conditions, cystic fibrosis is the one which had a link between alpha diversity and lung function (163). Another study showed the alpha diversity of gut microbiota could be a promising predictor for Alzheimer’s Dementia (AD), Schizophrenia, and Multiple Sclerosis (MS), but not for all neurological diseases (164) (Table 4).

##### 9.1.1.1 Metagenomics

Metagenomics is the study of the genomes in a microbial community and constitutes the first step to study the microbiome (165). Metatranscriptomics helps to identify the genes that are expressed. The sequencing of hypervariable regions and shotgun sequencing are technologies that enable the taxonomic classification of microorganisms from the DNA present in microbial communities. However, they are not capable of measuring what is actively expressed. Conversely, we advocate that metatranscriptomics is a “new” technology that makes the identification of the mRNAs of a microbial community possible, quantifying gene expression levels and active biological pathways. Furthermore, it can be also used to characterize symbiotic interactions between the host and its microbiome (166).

### 9.2 Mannitol-lactulose intestinal permeability test

Dysbiosis results in increased inflammation, elevated levels of zonulin, destruction of intestinal tight junctions, and intestinal permeability, which allow lipopolysaccharides (LPS) to leak into systemic circulation. LPS is a powerful endotoxin that causes chronic inflammation throughout the body. Chronic inflammation is associated with chronic diseases and the acceleration of biological aging (151).

Urinary excretion of lactulose and mannitol after oral intake is a good test for evaluating intestinal permeability and altered ratio indicates leaky gut syndrome (155) (Table 4).

### 9.3 Hydrogen or methane breath test

This common test is used to assess for small intestinal dysbiosis and also to assess the effectiveness of leaky gut treatment (167).

### 9.4 Identification of gut microbial metabolites: (metabolomics)

After taxonomic identification and genomic insights of microbiota and microbiome, we will focus on the functional capabilities and metabolomic characterizations using the technique of metabolomics. In simple terms it is functional readout of microbial activity (168, 169).

After that taxonomic identification, untargeted metabolomics profiling, and targeted metabolomics focusing on short chained fatty acids (SCFAs) analysis and others were done. Correlations between SCFAs and gut microbiota were also examined. Microbiome derived metabolites, such as lipopolysaccharides, SCFAs, secondary bile acids, or tryptophan-related metabolites play a role in the pathology of dysbiosis and can be measured from CSF (Cerebrospinal Fluid), plasma, urine, feces with NMR (Nuclear Medicine Resonance) spectroscopy analysis to measure quantitative metabolomics (170, 171) (Table 4).

Gut microbiota can function like an endocrine organ with bioactive metabolites like SCFA, trimethylamine N-oxide (TMAO), tryptophan metabolites (TRP) which can circulate in the human blood and be delivered to different target tissues. Trimethylamine N-oxide, p-cresyl sulfate and indoxyl sulfate have pro-inflammatory effects and may contribute to chronic inflammatory diseases. Tryptophan and its metabolites, indole acetic acid and indole-3-propionic acid, have been reported to enhance sensitivity of chemotherapy against cancer. To treat certain chronic diseases, a strategy using gut microbiota derived metabolites may be helpful.

### 9.5 Selected targeted metabolomics-measurement of SCFA

Three major SCFAs are acetic acid, propionic acid, butyric acid, and two less abundant SCFA are valeric acid and caproic acid. They are produced in the large intestine through the anaerobic fermentation of indigestible carbohydrates (172, 173). These microbial by-products can be measured using gas chromatography (156) and more specifically, gas chromatography–mass spectrometry can analyze SCFA in stools.

### 9.6 Trimethylamine *N* oxide

Carnitine and choline are commonly found in red meat and eggs, which were once thought to be semi-essential nutrients for the human body. However, these nutrients can be utilized by microorganisms in the gut to produce trimethylamine (TMA) as a byproduct. The TMA absorbed from the gut is then oxidized into TMAO in the liver and has proven to be a strong risk factor for cardiovascular disease (CVD) (174). Biomarker TMAO plays a role in cardiovascular disease, renal disease, type II diabetes and colorectal cancer (174).

Resveratrol may reduce the level of plasma TMAO and help in treating atherosclerosis in an animal study by acting like a prebiotic (175). Oral carnitine challenge tests are used to measure metabolites after gut microbial fermentation and to help identify TMAO-producer phenotype (158). Other gut metabolite biomarkers could be relevant to prodromal disease. Urolithins are anti-inflammatory metabolites produced from some dietary polyphenols by specific gut microbial ecologies (urolithin metabotypes) and have been proposed as biomarkers of gut microbiota composition and functionality (159). Thus, trimethylamine-N-oxide, short-chain fatty acids, 3-indoxyl sulfate, *p*-cresyl sulfate, secondary bile acids, hippurate, human β-defensin-2, chromogranin A, secreted immunoglobulins, and zonulin may serve as biomarkers for metabolite profiling with diagnostic suitability for dysbiosis and diseases (176).

### 9.7 Tryptophan metabolites

Tryptophan (TRP), the essential amino acid obtained from diet, is mainly metabolized through the kynurenine (KYN) pathway and it plays a role in different metabolic disorders. The gut microbiome can convert tryptophan into indole, and its derivatives, which can contribute to GI function, inflammation, antioxidation, and immune system regulation. Disorders in tryptophan metabolism can impact various diseases such as irritable bowel syndrome, colitis, depression, Alzheimer dementia, schizophrenia, and Parkinson disease. There is growing research about tryptophan metabolism disruption in neoplastic diseases, such as colorectal, liver, lung, and breast cancer (177). High-performance liquid chromatography-mass spectrometry, and gas chromatography–mass spectrometry can be used to measure tryptophan metabolites (178).

In conclusion, metagenomics and metatranscriptomics data are generated using sequencing data, whereas metabolomics data is analyzed using liquid and gas chromatography techniques, mass spectrometry (MS) and nuclear magnetic resonance (NMR) techniques. Integrating all metagenomics, metatranscriptomics, and metabolomics—would provide a complete picture from genes to phenotype (179).

From the authors point of view, doing CDSA and identification of gut microbial metabolites as the starting workup for dysbiosis and the next step is to use tests better than the taxonomic indicators to define microbiomes in health and disease.

### 9.8 Microbiome health index

Microbiome Health Index (MHI) was developed by Blount et al. to diagnose post-antibiotic dysbiosis. It is a promising biomarker of post-antibiotic dysbiosis and subsequent restoration of microbiota (180).

## 10 Genetics of microbial dysbiosis (non-modifiable risk factor)

There are a variety of factors that can contribute to alterations and differences in the gut microbiome seen with individuals. A study by Zoetendal et al. compared adult monozygotic twins to their unrelated marital partners and found that there were greater similarities between the gut microbiome among the monozygotic twins; this was hypothesized due to the influence of their genotype on the microbial diversity (181). Another interpretation of this was that the microbial similarities were due to the twins having a shared mother (181). Another study found that marital partners had different microbial communities colonized in their ear canal, however within families there were common dominant bacterial species (182). At this time, there is emerging evidence that there may be an interplay between host genetics and the gut microbiome, however the mechanisms are not completely understood.

In a genome-wide association study of 7,738 patients (from the Dutch Microbiome Project), the authors examined 207 taxa and 205 pathways and found a significant signal (*p* < 1.89 × 10^−10^) near the Lactase (LCT) and ABO genes that were associated with multiple microbial taxa and pathways (183). In particular, there were able to narrow down an association with *Bifidobacterium adolescentis* at the LCT loci and *Bifidobacterium bifidum,* and *Collinsella aerofaciens* at the ABO loci. Animal studies in pigs have found that a deletion at the ABO locus, that inactivates the ABO acetylglucosaminyltransferase (enzymes in glycoprotein biosynthesis), led to a change in the porcine microbiome composition (184). The study by Lopera-Maya et al. also found 22 other loci that may have an association with microbial taxa and pathways and be correlated with trait heritability, however a larger sample size is needed to further explore the role of host genetics on the gut microbiome (183).

Using metagenomic sequencing a genome-wide analysis using 1,514 subjects was done and found 9 loci with microbial taxonomies and 33 loci with microbial pathways and gene ontology terms (*p* < 5 × 10^–8^) (185). It was found that LCT single nucleotide polymorphisms (SNP) with the *Bifidobacterium* genus (*p* < 3.45 × 10^–8^) may in fact be a gene-diet interaction that can influence the abundance of *Bifidobacterium* (185). Other investigations looked at SNP-based heritability and used microbiome genome wide association to determine host genetic variants related with the gut microbiome. The group of Xu et al. found that Saccharibacteria could lead to a decreased serum creatinine concentration and potentially increase the estimated glomerular filtration rate through the interplay between host genetics and the gut microbiome (186).

## 11 Management of dysbiosis including risk factor modification

Various treatments can be used in managing dysbiosis and diet is an important step to improve dysbiosis (Tables 5, 6). Addressing the risk factors for dysbiosis, like avoiding medications that cause dysbiosis, stress management, avoiding ultra processed foods and alcohol, can help in the management.

**Table 5:** Management strategies of dysbiosis.

Classification	Method	Mechanisms of action
Direct repopulation	Fecal microbiota transplant	A method of repopulating the gastrointestinal tract with beneficial bacteria directly (187)
Gut biotics	Probiotics	Live microorganisms that can provide health benefits and are designed to restore the beneficial bacteria of the gut (188, 189)
	Prebiotics	Compounds found in food designed to promote the growth of beneficial microorganisms of the human gut (190)
	Synbiotics	Refers to food or dietary supplements that consist of both probiotics and prebiotics (191)
Diet/Food modifications	Fermented foods	Fermented foods may play a role in health benefit through the nutritive alteration of the ingredients, modulation of the immune system, and the presence of bioactive compounds. By modulating the gut microbiota composition and activity they can affect intestinal and systemic function. Ingestion may help intestinal barrier function along with the production of metabolites inhibiting the uptake of pathogens (192)
	Fiber rich foods	High-fiber diets have the ability to positively alter the microbial intestinal composition by promoting the growth of more beneficial bacteria, such as Prevotella and Bacteroides, while shifting away from Firmicutes (193). Dietary fiber can also selectively increase SCFAs producing bacterium abundance (194)
	Mediterranean diet	This diet is generally described as having a greater focus on minimally processed fruits and vegetables with the inclusion of pulses (e.g., Chickpeas, lentils), nuts, seeds, and fish in relative abundance. The diet itself has also been associated with improvement in microbiome composition and diversity which can lead to lower risk of gut dysbiosis (195, 196)
	Ketogenic diet	This diet focused on a considerable limitation of carbohydrate sources to promote ketone body production. These ketones bodies may lead to an impact on energy metabolism and impact on the microbiome influencing bacteria taxa, richness and diversity (197)
Microbial by-products	Metabolite treatment	The byproducts of the gut microbiome or even probiotics are highly bioactive and are sometimes called “postbiotics” (198). Some common metabolites are SCFAs, which are a fuel source for colonocytes and can help maintain the gut barrier and inhibit pathogenic microorganism proliferation due to acidic pH condition. Specific SCFAs, such as resveratrol, a phytoalexin, can decrease plasma TMAO (which is a risk factor for CVD) (175)

**Table 6:** Selected studies involving dietary interventions and dysbiosis.

Intervention	Type of study	Outcome	References
Probiotic	Human Meta-analysis *N* = 32 studies included	Subjects were found to have decreased blood glucose and HbA1c with increased HDL levels. Study suggests that probiotics could be a supplementary therapeutic approach in type 2 diabetes mellitus for dyslipidemia and metabolic control.	(199)
Artificial sweeteners	Human Cross-sectional Total = 31 participants Aspartame NC = 24 Aspartame C = 7 Acesulfame-K NC = 24 Acesulfame-K C = 7 Consumer of both = 20	Of the subjects, the aspartame and acesulfame-K consumers did not show any difference in the median bacterial abundance when compared to the non-consumers. There was an overall bacterial diversity difference in the aspartame (*p* < 0.01) and acesulfame-K (*p* = 0.03) consumers.	(200)
Various gut biotic (prebiotic, probiotic, or synbiotic)	Human Meta-analysis *N* = 38 studies included	Those receiving a gut biotic had lowered FBG (*p* < 0.01) and insulinaemia (*p* < 0.01) with increased HDL levels (*p* < 0.01). There was a reduction in HbA1c, but not statistically significant and no change to LDL levels. The use of gut biotics showed some improvement with metabolic variables and they may serve as a potential adjunct in treatment to help improve metabolic outcomes.	(201)
Fermented milk with *Lactobacillus acidophilus* La-5 and *Bifidobacterium animalis* subsp *lactis* BB-12	Human Double-blind, placebo-controlled Control = 23 Probiotic = 22	Individuals receiving the probiotic containing fermented milk has a lower HbA1c (*p* = 0.06). The control group (fermented milk alone), showed a reduction in interlukin-10 (*p* < 0.001) and both groups having reduced TNF-α and resistin. This suggested the fermented milk may have a role in metabolic changes through decreased inflammatory cytokines.	(202)
Probiotic (*Streptococcus thermophilus*)	Human *N* = 20 health Caucasian women	Individuals receiving a cream containing *S. thermophilus* showed an increase in stratum corneum ceramide levels after 2 weeks of application. This helped improve lipid barrier and increase resistance to age-associated xerosis.	(203)
Probiotic (multi-strain)	Human Randomized, double-blind, placebo-controlled Placebo = 18 Probiotic = 17	Asthmatic patients receiving the probiotic for 8-weeks had improved FEV and FVC with reduced levels of interlukin-4 and Th2 cells. Authors concluded that probiotics can be used as an adjunct with standard asthma treatments.	(204)
Probiotic (multi strain)	Human Randomized, double-blind, placebo-controlled Control = 20 Probiotic = 20	Patient receiving a probiotic supplement showed reduction in Beck Depression Inventory scores (*p* = 0.001). There were also lower serum insulin levels (*p* = 0.03), serum nightly CRP levels (*p* = 0.03), and homeostasis model assessment of insulin resistance (p = 0.03) in the probiotic group. There were no significant changes to fasting plasma glucose or lipid profiles.	(205)
Probiotic (*Lactobacillus casei* Shirota)	Human Randomized, double-blind, placebo-controlled pilot study *N* = 39 chronic fatigue syndrome patients	Patients with chronic fatigue syndrome receiving the probiotic treatment had reduced anxiety symptoms (*p* = 0.01) based on Beck Anxiety inventory compared to the control group. Those taking the probiotic were found to have increased levels of *Lactobacillus* and *Bifidobacteria* compared to controls.	(206)
Probiotic (multi strain)	Human Randomized control trial Placebo = 26 Probiotic = 26	Patients admitted to hospital for an acute mania, who received a probiotic treatment, had a reduced length of stay (*p* = 0.017) and rehospitalization (*p* = 0.007) compared to the control group. Authors felt that the use of a probiotic was well tolerated with low side effects that it may serve as an adjunct in the treatment of mania and other mood disorders.	(207)
Artificial sweetener	Mice Experimental Placebo = 5 Neotame = 5	Mice receiving Neotame had higher concentrations of cholesterol (*p* < 0.05) and fatty acids (*p* < 0.05) in fecal samples with a reduction in alpha diversity and altered beta diversity. It is suggested that the artificial sweetener has negative effects on the gut microbiome of mice and lead to a perturbation of the gut microbiome.	(208)
Functional fiber and metformin	Rats Experimental Placebo = 11 PGX = 11 Cellulose/MET = 11 Cellulose/S/MET = 11 PGX/MET = 11 PGX/S/MET = 11	Zuker diabetic fatty rats receiving PGX + MET or PGX + S/MET had reduced glycemia compared to controls (*p* = 0.001) with the HbA1c being lower in PGX + S/MET compared to all treatment options (*p* = 0.001). The use of a functional fiber (PGX) may contribute to the enhancement of metformin and metformin with sitagliptin function when co-administered. Authors feel this may have implications in the treatment type 2 diabetes.	(209)

C, Consumer; FBG, Fasting blood glucose; FVC, Forced Expiratory Volume; FVC, Forced Vital Capacity; HbA1c, Hemoglobin A1C; HDL, High-density lipoprotein; LDL, Low-density lipoprotein; NC, Non-consumer; PolyGlycopleX, functional fiber; MET, Metformin; S/MET, Sitagliptin and Metformin, Th2, T Helper 2; TNF-α, Tumer Necrosis Factor-alpha.

### 11.1 Food and food products

#### 11.1.1 Dietary interventions-diet/food modifications

Various diets have been examined in relation to their impact on the human microbiome.

##### 11.1.1.1 Fermented foods

Fermented foods are unique products that have many potential benefits that range from food safety to human health. Increased shelf life and stability of foods is a long-standing safety benefit of the fermentation process (210). Various methods to obtain fermented foods include spontaneous fermentation, specific starter culture use, and back slopping (utilization of previously fermented foods to start fermentation in a new batch) (192, 211). Fermented foods have the capacity to contain probiotic cultures that could directly confer potential human health benefits. It is important to consider various factors, including the number of live cultures present at the time of food consumption, as well as the specific strains present within the food (192). Other elements, including food matrix, packaging, food formulation and others can have an impact on the potential of these foods to benefit human health. To be considered a fermented probiotic food, various thresholds for consideration need to be met as noted in the International Scientific Association for Probiotics and Prebiotics (ISAPP) consensus statement from 2021 (192). Some potential benefits of these foods can include: displacement of pathogenic bacteria within the gut through microbiome compositional change, alterations to the digestibility/tolerability of foods (examples include reduced concentrations of phytates, lactose and fermentable sugars), metabolite benefits directly related to immune function (211).

##### 11.1.1.2 Plant-based fiber rich foods

A reduction in opportunistic bacteria and inflammatory bacteria were seen, along with an increase in good gut bacteria and their metabolites with a plant-based dietary approach (193). Subjects in an interventional pilot study consumed red beet root juice over 14 days showed changes in gut microbiome with statistically significant increases in *Akkermansia muciniphila* and decreases in *Bacteroides fragilis* potentially conferring metabolic benefits and possible reduction in the risk of diabetes and obesity. Statistically significant increases in some SCFA were also observed in this pilot study with isobutyric and butyric acid that may support those metabolic benefits (194). There are various studies examining orange juice and possible benefits to the gut. One study with functional orange juice showed growth of emerging probiotics such as *Bacteroides xylanisolvens* and decrease in other strains, such as *Clostridia* sp. Therefore, this prebiotic orange juice may enhance gut microbiota composition and be a potential functional food (212). In another human study examining the intake of blood orange juice, significant changes were seen regarding SCFA production (particularly propanoic acid and isobutyric acid) and improved cardiometabolic risk factors (213). An animal study compared two orange juices with 100% fruit juice (high sucrose and flavonoids) and fruit beverage (higher glucose and fructose) being offered to rats. Of note, the rats offered the 100% orange fruit juice showed improved microbial diversity with altered Firmicutes/Bacteroidetes (F/B) ratio (decrease) and insulin resistance improvement while the fruit beverage group showed no diversity change with an increased F/B ratio (214). Whole fruit in themselves can have considerable impacts on the microbiome with implications to GI transit time and constipation. The exact constituents responsible and the most ideal fruit type remains to be determined (215). In a systematic review and meta-analysis on different fruits, Huo et al. found kiwi fruit had a predominant effect on microbial culture amounts as well as improvements in functional constipation (216). Other studies showed that a vegan diet rich in fiber will increase SCFA and inhibit pathogenic bacterial colonization (217, 218).

##### 11.1.1.3 Mediterranean diet

The Mediterranean Diet has been examined more broadly in relation to health and the microbiome (195, 196). This diet is generally described as having a greater focus on minimally processed fruits and vegetables with the inclusion of pulses, nuts, seeds, and fish in relative abundance. Meat is included, although there is a reduction in frequency of this with particular limitation to processed meat, and foods rich in saturated fatty acids. Polyunsaturated fatty acids (PUFAs), monounsaturated fatty acids (MUFAs) with special focus on olive oil, phenolic compounds, omega 3 fatty acids, fiber and low glycemic index foods tend to be consumed in higher amounts as compared to a “Western Diet.” The implications of the above include a reduction in the risk of cardiovascular disease, diabetes, metabolic complications, cancer, inflammatory conditions among other health concerns (219–221). The diet itself has also been associated with improvement in microbiome composition and diversity which can lead to lower risk of gut dysbiosis. From the Mediterranean Diet, the specific constituents that lend themselves to health benefits include: a variety of minimally or unprocessed whole grains/cereals, legumes, a variety of produce with vegetables and salads, dried fruit, nuts/seeds, honey, and olive oil. Low to moderate consumption of poultry, eggs, fish, wine, unprocessed or minimally processed cheese and yogurt also play a role while red and processed meats are consumed in very low frequency. There is evidence to support various microbiome impacts from this diet with increased microbial diversity, and increases in the abundance of *Bacteroides, Prevotella, Lactobacillus, Faecalibacterium, Clostridium, and Oscillospira*. In contrast a decrease in the abundance of Firmicutes is noted (196).

##### 11.1.1.4 .Western diet

A Western Diet generally is defined as a diet that has an abundance of processed foods leading to increased intake of salt, saturated fat (possibly trans fats), and added sugars. Along with this, there is generally a reduced intake of fiber rich foods, whole grains, and fish. The consequences of this leads to lower intakes of PUFAs, MUFAs, phenolic compounds, omega 3 fatty acids, fiber and low glycemic index foods. This dietary pattern has the potential to erode human health in many ways including the gut microbiome. With this dietary approach increased opportunistic bacteria and inflammatory markers are seen with gut dysbiosis (221).

In an animal study, comparing Mediterranean diet (MD) to Western diet (WD) there was an abundance of mammary gland *Lactobacilli* in monkeys who take MD with a resulting increase in bile acid metabolites and decrease in reactive oxygen metabolites (221). Another study in humans showed subjects who adhere to MD were found to have higher levels of SCFA (222).

##### 11.1.1.5 Ketogenic diet

This diet has also been examined regarding its impact on human health as well as the microbiome itself. A Ketogenic diet typically has a considerable limitation in the amount of carbohydrates consumed with diets containing 20–50 g per day or less (5–10% energy intake). The purpose of this is to promote ketone body production (acetone, beta-hydroxybutyrate, acetoacetate) to be used as a fuel source as opposed to glucose impacting the microbiome and host metabolism (197). Regarding the microbiome, some animal and human studies have shown positive impacts (re-shaped gut microbiome and biological functions) and negative impacts (decreased variability in gut bacteria with increased pro-inflammatory strains) (223). It is possible that the modified gut microbiome may be critical to potential outcomes in relation to the ketogenic diet as seen in seizure management (224). Complimentary dietary modifications such as the inclusion of prebiotics, probiotics, fermented foods and others may minimize some potential drawbacks that the ketogenic diet may have on the microbiome as noted in this study (223). There is some potential promise for treatment or prevention of dementia with the ketogenic diet although human studies are few and in early stages (197). A review article by Dowis et al. points out that the ketogenic diet may have therapeutic benefits “helping with weight loss, improving lipid markers for cardiovascular health, healing a disrupted microbiome, improving epigenetic markers, reversing diabetes, or reducing the need for medication, and improving responses to cancer treatments.” But the article stressed the need for well-designed randomized controlled trials that should be done to confirm the therapeutic possibilities provided by this dietary intervention (225). It is important to highlight the relatively complicated nature of this diet in relation to more conventional dietary approaches in order to achieve ketosis where ketone bodies are promoted as an energy source. Some of the possible complications of this dietary approach can include nausea, vomiting, changes to satiety along with implications to bone mineral density, hepatic function, pancreatic function, blood glucose management, cardiovascular disease risk among other health concerns (226). These impacts do bear careful consideration prior to long term ketogenic diet implementation.

##### 11.1.1.6 Gut biotics

###### 11.1.1.6.1 Probiotics

Probiotics (such as *Bifidobacterium* and *Lactobacillus*) and prebiotics are known to improve gut health and restore bacterial gut balance to achieve eubiosis. There is some evidence that probiotics have been shown to alleviate functional gastrointestinal symptoms (FGID) which is commonly seen in dysbiosis (227).

While most probiotics show safety and recovery efficacy, the impacts in relation to disease improvements are statistically marginal (188). However, typical probiotics are not applied to specific diseases. Therefore, the selection and detailed description of new and disease-specific next-generation probiotics (NGP) are crucially necessary (188). NGP are individual bacterial strains through gene sequencing and bioinformatics tools. They are designed to better understand colonization, efficacy and safety of the probiotic bacteria (188, 189).

Nanoprobiotics and nanoprebiotics represent promising future strategies to target dysbiosis (228). Durazzo et al., showed in their meta-analysis that probiotics showed improvement with body weight in overweight individuals and improvements in various metabolic diseases including fatty liver and type 2 diabetes mellitus (229, 230).

Probiotics are shown in animal studies to help with wound healing (231). This might happen through the “brain-intestine-skin axis” by improving systemic immune response and affecting peripheral tissue response (232).

Since the strains introduced by probiotic intake may not colonize the gut permanently, probiotics may need to be taken periodically in order to sustain their benefits, but more research is needed in this angle. Various methods for probiotic foods to exert their actions exist as included in the ISAPP consensus statement (233, 234).

Probiotics may not be safe for all individuals. In immunocompromised or critically ill people, probiotics can increase opportunistic infections and so a risk benefit assessment should be done before recommending these products (235).

###### 11.1.1.6.2 Prebiotics

To be considered as a prebiotic, a food must provide a benefit directly to microorganisms that can improve human health (190). There are many potential food products that can meet this definition including fruits, vegetables, pulses, tubers, whole grains, and sourdough bread. Some caution is needed in individuals with inflammatory bowel disease and other digestive concerns. It is of value to consider increasing these foods in incremental amounts to limit digestibility issues and to improve tolerability. Other factors such as activity and hydrational status will also have considerable impacts in this regard. Dietary fiber has been shown in randomized controlled trial (RCT) to promote the growth of SCFAs producing bacteria which may impact type 2 diabetes management (236).

###### 11.1.1.6.3 Synbiotics

Synbiotic foods are an intentional combination between a prebiotic food source and probiotic microorganism (191). It is important to emphasize that both of these components are required to confer human health benefit. Two definitions of synbiotic foods have been considered. Complimentary synbiotics are foods that contain both a prebiotic and probiotic food component that work independently of each other to benefit human health. Another category to consider include synergistic synbiotic foods which also contain prebiotic fibers and probiotic microorganisms. The distinction here is that the prebiotic substrates must be selectively chosen to directly nourish the live bacterial cultures being included in the same food product with human health benefit as a result (191). The intentional prebiotic and probiotic combination can multiply potential health benefits to the host organism beyond impacts that could be reasonably expected from either component taken alone. The possibility of harnessing benefits that are greater than the sum of its parts poses a very intriguing possibility to human health improvements that can include microbiome modulation, and immune impacts to name a few (237).

##### 11.1.1.7 Foods to avoid to improve dysbiosis

###### 11.1.1.7.1 Processed foods

When the natural state of a food is changed for a specific reason, this can be considered a processed food. Some typical purposes of food processing include shelf stability, enhancements to food safety, improvements to food palatability/taste, and increase in nutritional value. To achieve these purposes foods may be pasteurized, canned, chemically altered, fermented, frozen, and dried, among other techniques.

Processed foods can be defined in various ways but are perhaps best defined via the NOVA classification system which divides food products into four groups based on the degree of food processing. NOVA Classifications: (1) Unprocessed or minimally processed foods, (2) Processed culinary ingredients, (3) Processed foods, (4) Ultra-processed foods (238). Some of the examples for (1) Unprocessed or minimally processed foods: Milk, Eggs, Carrots, Broccoli, Potatoes, Chicken, Oats, Rice, Dried Pulses, Unsalted nuts, (2) Processed culinary ingredients: olive oil, sugar, honey, salt, (3) Processed foods: canned tuna, canned pulses, salted/flavored nuts, tomato paste, homemade bread, wine, (4) Ultra-processed foods: chocolate, candies, potato chips, ice cream, pre-made pizza/burgers, carbonated soda beverages (238).

There is robust evidence to support the harms of ultra processed foods to human health with connections between ultra processed foods and dysbiosis which highlights the importance of identifying these foods within individual diets (239–242). The intake of ultra-processed foods can help promote a microbial environment that tends toward inflammation and oxidative change that increases the risk of gastrointestinal health concerns like inflammatory bowel disease, neurodegenerative diseases, and metabolic health consequences including obesity and beyond (239–242). The specific dietary components of ultra processed foods that can relate to human gut microbiome harm include higher intake amounts of sugar, fat, salt and food additives with reduction in dietary fiber, polyunsaturated fatty acids and phenolic compounds. Impacts to the microbiome seen from these constituents included an increase in the genus phyla Firmicutes with reductions in Bacteroidetes. Increases in *Lactobacillus*, *Faecalibacterium* of the *Clostridium* cluster IV are seen with ultra processed foods. Depletions in dietary fiber led to reductions in Bifidobacterium and some *Clostridium* subgroups (*Roseburia* and *Eubacterium rectale*) (241).

###### 11.1.1.7.2 Food additives/preservatives

There is growing evidence to show that food additives and preservatives also likely play a role in disturbing the gut microbiome (243). Non-caloric sweeteners, emulsifiers, antimicrobial preservatives, food colorants and other additives can promote dysbiosis leading to many potential consequences which may include impairments to glucose metabolism, inflammation and/or increased chronic disease risk (244, 245). The impact of food additives to the microbiome can be vast with impacts to gut microbiota across various species including Firmicutes, Bacteroidetes, Barnesiella, Prevotella, Ruminococcaceae, and Bifidobacterium. Whether these constituents are decreased or increased does seem to vary widely based on the food additive being studied as noted by Song et al. (246) and Zhou et al. (247).

A human randomized control trial study was done showing that emulsifier use (Carboxymethylcellulose) impacted the microbiome with decreases in *Faecalibacterium prausnitzii* and *Ruminococcus* sp., and increases in *Roseburia* sp. and *Lachnospiraceae* (248).

In another human trial examining microbiome impacts of food additives to human fecal samples, sodium benzoate increased the amounts of *Bifidobacterium* while sodium sulphite decreased *Bifidobacterium* while increasing *Escherichia coli* and *Shigella* (249).

It is clear that these dietary components have a definitive impact on the gut microbiome with further human studies needed to delineate health consequences.

### 11.2 Lifestyle changes

Smoking, alcoholism, physical activity, stress and sleep deprivation contribute to dysbiosis. It has been found that cigarette smoking can lead to intestinal and microbial dysbiosis (64, 250). Other studies have found that smoking cessation improved intestinal dysbiosis (251, 252). A study by Leclercq et al. found that with chronic alcohol consumption there are changes in the gut microbiome and decreased intestinal barrier integrity which can lead to increased depression, anxiety, and craving through the microbiome-brain-gut axis (253, 254). Muthu et al. in their study showed subjects with chronic alcoholic consumption had lower percentage of *Clostridia, Bacilli* and *Bacteroidetes* whereas a higher percentage of Gammaproteobacteria (254). A meta-analysis showed alcohol can affect the microbiome derived metabolites like neurotransmitters which are associated with mood and behavioral disorders secondary to alcohol intake (256). Alcohol is shown to damage the microbiome but with abstinence, a reduction in gut dysbiosis can be seen (253).

Disruptions in sleep can have an impact on the gut microbiome (257), whereas improvements with sleep lead to positive changes in microbial diversity (258). Though more research is needed, a meta-analysis found that patients using a gut biotic reported better perceived sleep health (259). Lifestyle can also have an impact on gut microbiome health and diversity. Individuals with a more sedentary lifestyle were found to have less microbial diversity and more bacterial species associated with disease, such as *Escherichia coli* (260). In comparison, individuals that have a more active lifestyle had a richer bacterial diversity and reduced dysbiosis with more SCFA producing bacteria (260). When looking at the role of stress on the gut microbiome, psychological stress can lead to altered bacterial composition (261). In stressful events, the Hypothalamic Pituitary Adrenal (HPA) axis becomes temporarily active leading to the release of various hormones. With prolonged activation, this can lead to heightened inflammation that can impact gut barrier permeability and lead to dysbiosis (262).

### 11.3 Impact of food processing technology on dysbiosis

#### 11.3.1 Microwave treatments

One of the major factors that can influence the gut microbiome is our diet. Along with this, emerging research is highlighting that it is not just the food items, but the ways in which we prepare and process our food that can impact the microbiome. In particular, the use of microwave technology has been linked to the utilization of dietary fibers by the gut microbiome.

Microwave treatments may provide a beneficial impact to the fermentability and health impact of dietary fibers leading to an improvement in SCFA production and impacts on bacterial changes (263, 264). Microwave impact on specific dietary fibers is noted with some improvement to fermentability although impacts to whole meals remain to be seen. A study using microwave treatment in combination with enzymatic processing showed an increased availability of dietary fiber. This processing promotes an increase in the F/B ratio. Overall, this processing technique increases the availability of insoluble fiber for fermentation (265).

### 11.4 Microbiome-based therapies

#### 11.4.1 Fecal microbiota transplantation

Fecal microbiota transplantation (FMT) is the transfer of fecal bacteria from a healthy donor to a recipient. The purpose of this is to repopulate the recipient’s GI tract with beneficial bacteria. It is most notably used in patients with *Clostridioides difficile* infection, but the principle may be applied to microbial dysbiosis to help restore healthy bacteria. For the management of recurrent *C. difficile,* the use of FMT has been approved in the USA as the infection can occur 25–35% during index infections and up to 60% with recurrent cases (266). CDC (Centre for Disease Control) recommends microbiome sparing antibiotic Fidaxomicin as the first line therapy, which also helps to prevent recurrence. It also recommends microbiome therapeutics like Fecal Microbiota Transplantation (FMT) in recurrent *C. difficile* infection, which hope to reduce the dependence on antibiotics for recurrent infection (267). Safety concerns in different type of populations should be explored for FMT in future research studies. There have been various animal studies looking at the effects of mood and behavior through the use of FMT. One study took fecal samples from patients with depression and transferred them to germ-free mice. These mice began to exhibit more depressive-like behavior (268). When looking at some human studies, there are emerging case-reports of patients with a diagnosis of Alzheimer dementia showing improvement in memory and mood after receiving a FMT for a *C. difficile* infection (269, 270). A double blind RCT looked at patients with irritable bowel syndrome (IBS) who received a FMT and found a reduction of symptoms, such as fatigue, up to 3 months following treatment with reduction in the dysbiosis index (187). A recent meta-analysis looked at the efficacy of FMT in IBS and found the mode of delivery may have an impact on benefit, with colonoscopy and nasojejunal tube more impactful than oral capsules (271). At this time, further research is still needed about the role of FMT in the treatment of various diseases.

#### 11.4.2 SCFA

SCFA derived from indigestible carbohydrates can participate in the metabolism of bile acid (BA) and lipopolysaccharide (LPS) (272). SCFA has been shown to suppress the proliferation and induce apoptosis of tumor cells (273, 274). SCFA can also be used in the treatment of auto immune disorders (275). Probiotics, prebiotics and synbiotics can modulate the growth and metabolic activity of the microbiota. Use of prebiotics and probiotics that modulate local and systemic SCFA concentrations appears to be a promising therapy in infections (276). Recent preliminary evidence points out that SCFA has the potential for treating type 2 DM (277). More research is needed in this area.

SCFA may have a role in the management and treatment of chronic kidney disease owing to reduction in inflammation and oxidative stress (278). Valerate or valeric acid is another short chain fatty acid produced in small amounts during the fermentation of dietary fiber. This short chain fatty acid is depleted from the gut following antibiotics and restored with fecal microbiota transplantation. In a pre-clinical study valerate decreased the incidence of *C. difficile* in a mouse model of infection (279). In another study examining valeric acid level, it was noted that more depleted valeric acid amounts were present in ultra high-risk groups prior to conversion to schizophrenia and in those already with the mental health disorder. This suggests that valeric acid may be involved in the conversion to schizophrenia (280).

The benefits of anti-inflammatory impacts related to SCFA may even extend to the epithelium including treatment of various conditions such as psoriasis and acne (281). Mental health including epilepsy may even benefit from SCFA through various pathways including neurotransmitter impacts, the protection of the blood brain barrier, reduction of oxidative stress to neural tissue and downregulation of psychosocial stress (282) Studies have shown the role of SCFA in treating cancers, autoimmune diseases, infections, type 2 diabetes mellitus, chronic kidney disease, epilepsy and inflammatory skin diseases. Although SCFA impacts are quite encouraging across many health conditions, human studies in this area remains limited. More research and clinical trials are needed to reveal the therapeutic potential of SCFA.

#### 11.4.3 Postbiotics

Postbiotics are soluble components of microbial cells or their derived metabolites that can provide therapeutic benefits (198). Species other than those belonging to the traditionally safe genus *Bifidobacterium* or the family Lactobacillaceae, which could not be administered live due to concerns about their safety, have been explored as potential postbiotics (283).

## 12 Dysbiosis and related costs

The impact of microbial dysbiosis can lead to increased health care costs related to both acute and chronic conditions. In particular, antibiotic-associated diarrhea can lead to increased morbidity and lengthier hospital admissions, requiring more healthcare resources (284). In the United Kingdom, the resulting intensive care unit stays and need for readmission was speculated to cost £13,272.53 per patient with antibiotic-associated diarrhea (284). As well, patients with a *C. difficile* associated diarrhea often require extended hospitalization and multiple medical treatments, including laboratory tests. In the United States, data from 2014 found that patients with a primary *C. difficile* associated diarrhea would incur $24,205 USD in health care costs while a recurrent *C. difficile* associated diarrhea patient would require US$10,580 (284).

As previously noted, dysbiosis can influence many chronic diseases with considerable implications among these illnesses. Chronic health conditions are associated with increasing resource costs to society with the CDC indicating that “90% of the nation’s $4.1 trillion in annual health care expenditures are for people with chronic and mental health conditions”(285). Dietary and lifestyle approaches possess a great deal of promise to combat chronic conditions that may be influenced in considerable ways by dysbiosis and microbiome imbalance. Making use of these relatively non-invasive strategies seems prudent to minimize both health risks and societal costs.

## 13 Benefits and limitations to dysbiosis diagnosis and management

There is currently no specific method or gold standard technique to diagnosis microbial dysbiosis in a patient. To date, the use of a stool sample analysis is the most common way to interpret the state of a patients gut microbiome and if a potential dysbiotic state exists. By continuing to develop more specific tools and methods, such as microbial metabolite detection, a better comprehension of changes in the gut microbiome can be gained. From this, there may be further understanding in how the gut microbiome may play a role in the physiology and pathology of certain human diseases. Further, gene-level and bioactive microbial protein analyses of microbiome-disease is better than taxonomic analysis.

There remains certain limitation in our knowledge around the gut microbiome, including that there is no one consistent model that serves as a means to capture the phenotypic diversity and complexity of the microbiome. As well, the concept of an “ideal microbiome” has not been established. Thus, the beneficial bacteria for one individual may not serve the same benefit for another (286). As well, clear guidelines or protocol on the treatment of a dysbiotic state, as well as ways to maintain a healthy gut microbiome has not been established. Even through various lifestyle and dietary interventions, there may be a need for a more personalized therapeutic approach for the treatment of gut dysbiosis (286).

In recent years many publications have highlighted the role of microbiota and dysbiosis in different diseases. Like any other diseases, genetic, epigenetic, lifestyle and environmental factors play a role in the medical condition of dysbiosis. Systemic screening of microbiota and measuring metabolites is now possible. In recent years, there are many targeted studies investigating gut microbiome alternations in different human diseases. Abnormal metabolites levels have been linked to certain diseases. For example, trimethylamine levels are associated with cardiovascular disorders, bile acids like deoxycholic acid and lithocholic acids with colorectal cancer and SCFA butyrate with cognitive disorder. Use of simple supplemental therapies like probiotics, prebiotics, synbiotics with regular treatment can potentiate the effect or reduce the toxicity of treatment for diseases. Obviously more interventional study research is needed in humans. Role of diet in shaping microbiota is also changing the view of strategies of improving systemic and whole-body health. With microbiome-based therapies, dysbiosis can also be treated by transplanting bacteria or bacterial-derived byproducts (SCFA, post biotics) to ameliorate the microbiome and restore health. For wide spread use of these therapies more research is needed. Microbiome testing is still in its infancy and has limited value for day-to-day practice at this point. A new form of microbiome therapeutics is the evolving phage therapy.

Overall, with new emerging microbiome studies with different medical conditions, analyzing the microbiome with conventional methods of diagnosis and using the different strategies for the management of dysbiosis along with traditional management may improve healthcare, especially where conventional approaches have failed (287). Broad adoption by medical communities will help with the advancement of ways to treat diseases using the microbiome-based approaches.

## 14 Conclusion

Human gut microbiome in multiple studies has been shown to play an important role in health. Dysbiosis can be considered as a medical condition. Whole body dysbiosis causes imbalance in the composition or function of gut and non-gut related microbiome and can have a broad clinical presentation as a medical disorder from metabolic syndromes to cancer. Definition of normal gut microbiota has not been clearly defined so far. It varies between individuals based on genetics, food preferences, lifestyle, geographic and environmental factors. Much of the understanding about dysbiosis comes from animal studies. Considerable evidence from both animal and human studies has accumulated showing a clear link between the microbiome imbalance in diseases. Understanding the communication and pathways involved in these interactions are essential to improve our knowledge of dysbiosis, and our ability to treat or prevent dysbiosis. There are different interactions between the gut and different organs that regulate function, called the gut–organ axis. Understanding the microbiota–gut–organ-axis has opened the door for better appreciation of different disease pathologies and offers opportunities to study microbial therapeutics through the regulation of the microbiome. Dysbiosis can be seen as an initiation, perpetuation or outcome of diseases and can be the target for treating these conditions. It can affect any body part that has its own special ecosystem of microorganisms throughout the whole individual. Dysbiosis may have different impacts on different hosts depending on the nature of the dysbiotic community and underlying genetic predispositions for disease.

Gut microbiota metabolites like SCFA play a role in dysbiosis. Tests used to diagnose dysbiosis are the urine test, hydrogen breath test, comprehensive digestive stool analysis, intestinal permeability test, microbiome diversity test, and measurement of SCFA levels. Many medically necessary treatments including medications impact the microbiome.

Diet and lifestyle changes should be considered as a therapeutic approaches to improve dysbiosis. The microbiome may vary daily, weekly and monthly depending on diet and life style factors. Metabolites derived from gut microbiota like SCFA can play a role in the development of diseases. Certain diets can influence different gut bacteria and metabolites. Treating dysbiosis with lifestyle changes and diet modification (including avoiding ultra processed foods) can alter the gut microbiome composition and function. Diet and lifestyle alterations appear to be the most obvious, non-invasive, and immediate way of altering the microbiome composition and function. There are ongoing research and clinical trials in the field of microbial therapeutics. Identifying and reversing dysbiosis can be life-changing for many people. In a dysbiotic condition, dietary and lifestyle modifications, treatment with gut antibiotics, and, with more severe cases, faecal transplantation, are the interventions used to correct this state. There are only a limited number of human research studies to show this relationship. Further human studies are needed in this area to more clearly elucidate pathways, mechanisms and benefits to human health. Overall evidence at this point shows dysbiosis as a probable new therapeutic target in the management of diseases.

## Author contributions

KA: Conceptualization, Writing – original draft, Writing – review & editing. JM: Conceptualization, Writing – original draft, Writing – review & editing. TH: Conceptualization, Writing – original draft, Writing – review & editing.
